# Enhanced Sensing Behavior of Three-Dimensional Microfluidic Paper-Based Analytical Devices (3D-μPADs) with Evaporation-Free Enclosed Channels for Point-of-Care Testing

**DOI:** 10.3390/diagnostics11060977

**Published:** 2021-05-28

**Authors:** Jaehyung Jeon, Chanyong Park, Dinesh Veeran Ponnuvelu, Sungsu Park

**Affiliations:** 1 School of Mechanical Engineering, Sungkyunkwan University, Seoburo 2066, Jangan-gu, Suwon 16419, Korea; jjh4760.official@gmail.com (J.J.); cksdyd6348@naver.com (C.P.); vp.dinesh@gmail.com (D.V.P.); 2 Department of Biomedical Engineering, Sungkyunkwan University, Suwon 16419, Korea; 3 Institute of Quantum Biophysics (iQB), Sungkyunkwan University, Suwon 16419, Korea

**Keywords:** paper-based analytical devices, 3D printing, enclosed channel, small-volume sample, evaporation, point-of-care testing

## Abstract

Despite the potential in fabrication of microfluidic paper-based analytical devices (μPADs) for point-of-care testing (POCT) kits, the development of simple, accurate, and rapid devices with higher sensitivity remains challenging. Here, we report a novel method for 3D-μPAD fabrication with enclosed channels using vat photopolymerization to avoid fluid evaporation. In detail, height of the enclosed channels was adjusted from 0.3 to 0.17 mm by varying the UV exposure time from 1 to 4 s for the top barrier, whereas the exposure time for the bottom and side barriers was fixed. As a result, sample flow in the enclosed channels of 3D-μPADs showed lesser wicking speed with very scant evaporation compared to that in the hemi channels in the 3D-μPADs. The stoppage of evaporation in the enclosed channels significantly improved the gray intensity and uniformity in the detection zone of the 3D-μPADs, resulting in as low as 0.3 mM glucose detection. Thus 3D-μPADs with enclosed channels showed enhanced sensitivity compared to the 3D-μPADs with hemi channels when dealing with a small volume sample. Our work provides a new insight into 3D-μPAD design with enclosed channels, which redefines the methodology in 3D printing.

## 1. Introduction

**Figure 1:**
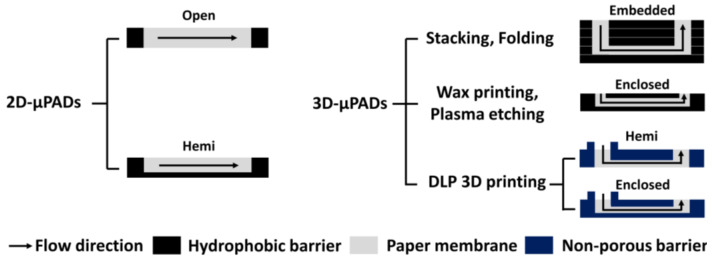
Cross-sectional views of different 2D- and 3D-μPADs classified by their channel type.

Over the past decades, many efforts have been focused on the development of microfluidic paper-based analytical devices (μPADs) as a platform for point-of-care testing (POCT) kits because they are inexpensive, simple, and easy to use [1,2,3]. 2D-μPADs with either open or hemi channels can be fabricated by simply forming hydrophobic or non-porous boundaries in paper (Figure 1) [4,5,6]. Despite this simplicity, sample solution evaporates in 2D-μPADs through their channels and some analytes thus cannot reach the detection zone, resulting in a low detection sensitivity [7,8,9].

To address this problem, researchers have proposed several methods for fabricating 3D-μPAD in which fluid can flow in both horizontal and vertical directions. Among them, the simplest one to complete 3D-μPADs is to stack a few sheets of paper with 2D hydrophobic barriers [10] or fold a sheet of paper with 2D hydrophobic barriers vertically [11] (Figure 1). Despite this relatively simple and easy fabrication, these 3D-µPADs often suffer from leakage of sample solution due to adhesion failure or contaminated channels by melted adhesive. To avoid these problems, researchers have developed several methods to fabricate 3D-µPADs with enclosed channels without extra assembly steps using either wax printing [12,13,14] or plasma deposition with etching [15,16]. The wax printing-based method is very useful for the rapid fabrication of enclosed channels. However, when they are stored for a long time, wax from their channel walls could be released, causing contamination in the channels [12]. The plasma deposition with etching-based method is free from the contamination issue. However, the etching step could change the pore size of the paper and still requires extra steps for sealing the channel with double-sided tape [16].

Most recently, we reported on a method for the fabrication of 3D-μPADs with hemi channels by separately printing the side and top barriers within a filter paper using vat photopolymerization [17,18,19] (Figure 1). In this method, a filter paper immersed in photocurable resin was exposed layer-by-layer with visible or ultraviolet (UV) light, enabling the channels and reservoirs fabrication both inside and outside the paper. Unlike the conventional stacking method [11] and folding method [12], our method has the advantage of fabricating 3D-μPAD without additional assembly using adhesive tape. However, our previous 3D-μPADs are still not free from the evaporation issue because of hemi channels on the bottom.

**Figure 2:**
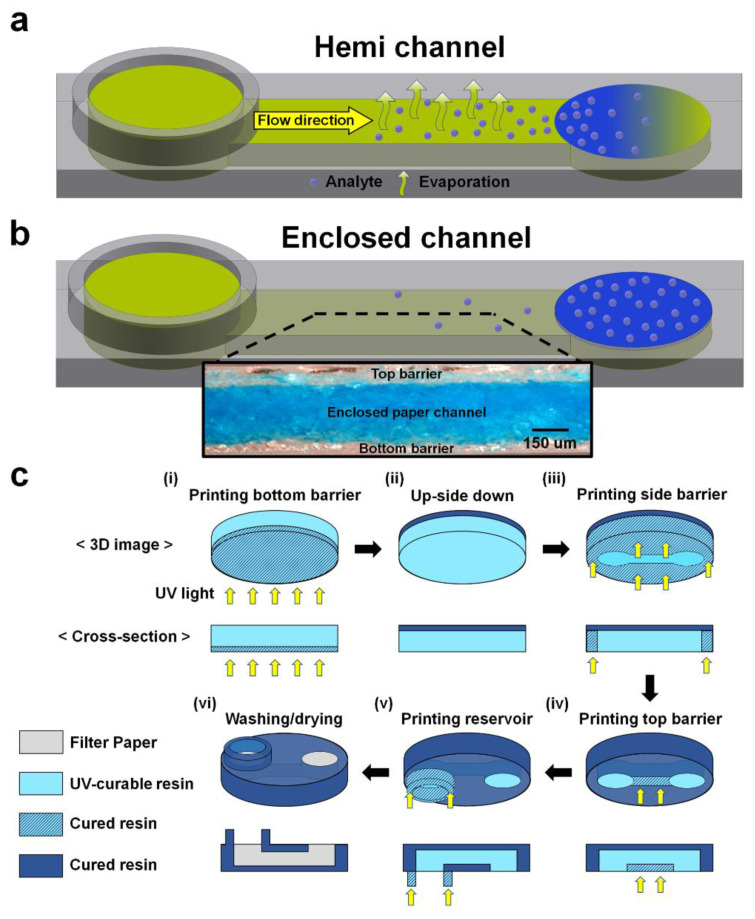
Schematics describing 3D models and printing steps of hemi and enclosed channels. 3D model of hemi (**a**) and enclosed (**b**) channels. (**c**) Vat photopolymerization steps to fabricate an enclosed channel in filter paper. The paper was soaked with the UV-curable resin before initiating printing steps (**i**–**vi**). Exposure times for the bottom, side, and top barriers and the reservoir were 1, 8, 1–4, and 20 s, respectively. Washing and drying steps (**vi**) were performed outside the printer.

Here, we report on an improved 3D printing method to fabricate enclosed channels. In this method, the 3D-μPADs with enclosed channels were fabricated by printing bottom, side, and top barriers in the filter paper in a sequential manner using a vat photopolymerization 3D printer (Figure 1 and Figure 2c). Unlike our previous 3D printing methods [17,18,19], all surfaces of the channel were exposed to UV light in order to fabricate an enclosed channel, which was surrounded with non-porous barriers. It took less than 10 min to fabricate 3D-μPADs, including washing and drying processes to remove un-reacted crosslinked photocurable resin and solvent. The channel height is inversely related to the UV exposure time of the top barrier. Flow inside the enclosed channels of 3D-μPADs showed lower wicking speed with less evaporation and contamination, compared to that in the hemi channels in the 3D-μPADs. Because of these promising advantages, detection signals of the enclosed channels were highly uniform in comparison with those observed in the hemi channels when glucose was colorimetrically detected via 3D-μPADs, thus reflecting the enhanced sensitivity.

## 2. Materials and Methods

### 2.1. Materials

D-(+)-Glucose, glucose oxidase (GOx) from Aspergillus niger, 4-aminoantipyrine (4-AAP), and horseradish peroxidase (HRP) were purchased from Sigma-Aldrich (St. Louis, MO, USA). N-Ethyl-N-(2-hydroxy-3-sulfopropyl)-3,5-dimethylaniline sodium salt monohydrate (MAOS) was purchased from Dojindo (Rockville, MD, USA).

### 2.2. 3D Printing of 3D-μPADs with Either Hemi or Enclosed Channels

To maximize the difference in evaporation, we designed top open hemi channels instead of the previously reported bottom open hemi channel (Figure 2a and Figure S1) [17]. The enclosed channels were designed to prevent evaporation of the sample as all sides of the channel were surrounded with 3D-printed barriers (Figure 2b). Patterns of the bottom barrier were designed in one layer, while patterns of the side and top barriers and a reservoir were designed in the other layer. The designs were drawn using the Student Edition of Inventor^®^ Professional (Autodesk Inc., San Rafael, CA, USA) and saved as different STL files. After that, the files were converted into slicer files by Slicer V1 (Carima, Seoul, Korea). The silicer files were finally printed into paper by a vat photopolymerization 3D printer (IM1, Carima). Vat photopolymerization parameters for enclosed channel fabrication are listed in Table 1.

Before printing, filter paper (qualitative filter paper: pore size: 6 μm, thickness: 390 μm from Whatman International Ltd., Maidstone, UK) was cut to fit the plate size (12 mm × 7 mm) of the 3D printer. Thereafter, the cut paper was soaked with UV-curable polyurethane resin (CFY044W, Carima) in a Petri dish for 10 s. It was moved to a tray filled with the resin and manually attached to the plate.

For printing, the bottom side of the paper was first exposed to UV at 405 nm for 1 s to print the bottom barrier (Figure 2c(i)). Then, the paper was turned upside down (Figure 2c(ii)), and its bottom side was exposed to UV for 8 s to print the side barriers (Figure 2c(iii)). After the side barriers were printed, the bottom side was exposed again for different times (1–4 s) to print the top barrier, depending on the enclosed channel height (Figure 2c(iv)). Finally, a sample reservoir of 2 mm height was printed from the bottom of the paper by exposing the bottom layer with UV for 20 s while moving the plate upward (Figure 2c(v)).

The paper was removed from the plate and was initially rinsed three times with 100% ethanol and rinsed again by spraying ethanol into the channel using a syringe (10 mL) without attaching a needle (Figure 2c(vi)). To completely remove the residual resin inside the channel, we carefully injected ethanol into the channel through a block (2 cm × 1 cm × 1 cm, *L* × *W* × *H*) of polydimethyl siloxane (PDMS) (Dow-Corning, Cortland, NY, USA) with a hole (3 mm) using a syringe without a needle. Finally, the paper was dried at 60 °C in an oven for 1 min.

The printing steps of the hemi channels with open top were similar to those of the enclosed channels, except for skipping the step shown in Figure 2c(iv). The hemi channels were washed three times with ethanol without the use of a syringe and the PDMS block.

### 2.3. Measurement of Height of Enclosed Channels

Enclosed channels were visualized by staining them with blue ink through the reservoir on 3D-μPADs (Figure 2c(vi)). Then, they were dried at 60 °C in an oven for 1 min. After drying, they were cut, and their cross-sections were imaged using a stereomicroscope (SMZ1500, Nikon, Tokyo, Japan) with a charge-coupled device (CCD) camera. The channel height was estimated by measuring the vertical distance of the ink-dyed area using ImageJ (NIH, Bethesda, MD, USA).

### 2.4. Observation of Crosslinked Resin in Hemi and Enclosed Channels

The crosslinked resin in hemi and enclosed channels was observed using a field-emission scanning electron microscope (FE-SEM) (JSM7500F; JEOL Ltd., Tokyo, Japan). Before imaging, the paper with channels was cut into a 5 × 5 mm sample. The surface of the sample was coated with iridium (Ir) for 15 min using an ion sputtering machine (Seron Technologies Inc., Uiwang-Si, Korea). The coated sample was attached to a stub of the SEM using double-sided conductive carbon tape. The SEM was operated at 15 kV, and the working distance (WD) was 8 mm. All images were captured at a magnification factor of 140 or 150.

### 2.5. Measurement of Sample Wicking Distance in Hemi and Enclosed Channels

Red ink was passed through hemi or enclosed channels by placing 30 μL of the ink onto a reservoir in 3D-μPADs. An image of a channel during the wicking was captured every 30 s using a tablet (Galaxy Tab S6, Samsung, Suwon, Korea). The wicking distance of the ink was analyzed using ImageJ. In the case of hemi channels, adhesive tape (1.5 mm in width) was attached to the entry part of the channels, preventing the fluid from flowing along the paper surface as a valve. The wicking distance was measured as the linear distance from the point where the reservoir started to the point where the ink flowed and measured three times per same channel height.

### 2.6. Analysis of Evaporation in Hemi and Enclosed Channels with Small Volume Samples

To maximize the evaporation effect between the two channels, we reduced and analyzed the volume of the sample. To observe the effect of evaporation on the wicking distance in each channel (15 mm in length, 0.21 mm in height), we passed 10 μL red ink through a hemi or enclosed channel, and its wicking was observed every 30 s. Wicking distance of both the hemi channel and the enclosed channel were measured three times.

### 2.7. Colorimetric Detection of Glucose in 3D-μPADs with Either Hemi or Enclosed Channels

3D-μPADs consisted of four hemi or enclosed channels (6 mm in length) connecting a sample inlet (4 mm in diameter and 2 mm in height) in the center with three detection and control zones (4 mm in diameter) in the periphery. For enzyme immobilization, 2.5 μL of a mixture containing MAOS (1 mM), 4-AAP (10 mM), HRP (1 mg mL^−1^), and GOx (10 mg mL^−1^) in PBS (pH 7.4) was dropped in the detection zone and incubated at 37 °C for 10 min [17,20]. Glucose with different concentrations (0–20 mM) was prepared by diluting a stock solution of d-(+)-glucose (3.7 mg mL^−1^) with PBS. The minimum volume (20 μL) of glucose, which is required to fully wet a hemi channel, was placed on the sample reservoir of the 3D-μPADs. Then, the 3D-μPADs were incubated at RT for 5 min until the color was generated on the detection zones. Images of the detection zones were captured using a stereomicroscope and analyzed with ImageJ.

Gray intensity in the detection zone was calculated by subtracting the mean gray intensity of the detection zone from that of the control zone. A standard deviation of the gray intensity in the detection zone was used to quantify the color gradient [21]. Mean gray intensity and color gradient in the detection zone were measured three times for each concentration. The LOD was calculated by multiplying the standard deviation of the negative control by three [18,22].

### 2.8. Statistics

Statistical comparisons of the results were analyzed with two-tailed Student’s *t*-tests when two cases were compared.

## 3. Results and Discussion

### 3.1. Fabrication of Enclosed Channel

**Figure 3:**
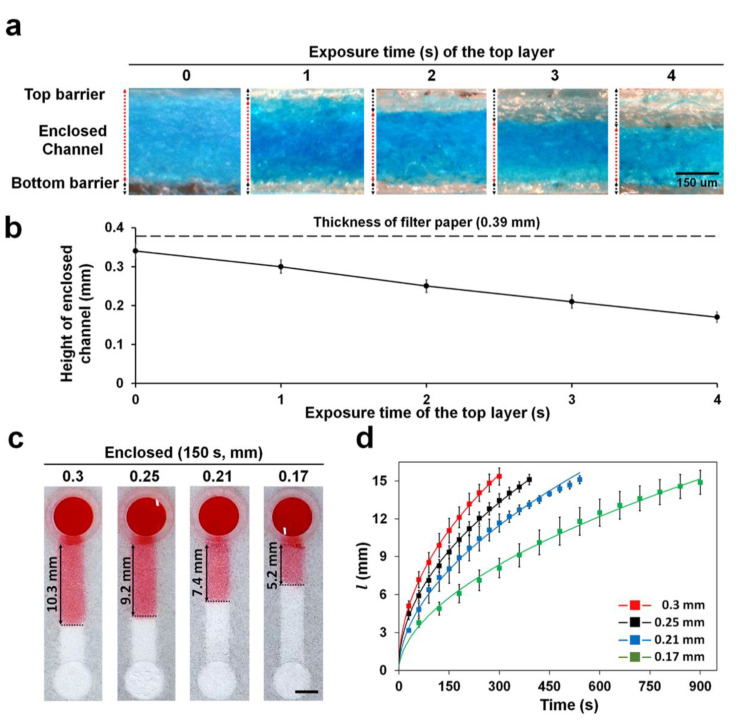
Height of enclosed channels in filter paper at different UV exposure times (0–4 s). (**a**) Cross-sectional views of enclosed channels stained with blue dye flowed from the reservoir. Bare filter paper maintained its hydrophilicity and was stained with the dye. (**b**) Enclosed channels with different heights by varying UV exposure times to print the top barrier in the paper once the bottom and side barriers were printed with UV exposure at 1 and 8 s, respectively. *n* = 3. (**c**) Image of water wicking along an enclosed channel. The image was taken 150 s after the water dropped on the reservoir. Scale bar: 3 mm. (**d**) *l* vs. time curve of water wicking along an enclosed channel with height varying from 0.17 to 0.3 mm. Sample volume: 30 μL, *n* ≥ 3.

The formation of the enclosed channel in the paper was confirmed by staining the channel with blue ink. The printed sides (top, side, and bottom) were left un-stained because of the hydrophobic property of the crosslinked resin; only the bare cellulose structures inside the channel, which is hydrophilic, were stained using the dye (Figure 3a). The height of the enclosed channels was easily altered by varying the UV exposure times for the top barrier while fixing the exposure time for the bottom and side barriers at 1 and 8 s, respectively (Figure 3a,b). The exposure time of the top barrier is inversely related to the channel height as depicted in Figure 3b. At exposure time higher than 4 s, the channel was often blocked by residual polymer that cannot be easily removed owing to the difficulty in passing solvent through the thin channel (data not shown). This indicates that enclosed channels can be fabricated as shallow as 0.17 mm. The coefficient of variation (CV) value of the height in all the enclosed channels was less than 7.8%, showing good reproducibility. This is the first study to report the fabrication of enclosed channels on the paper using 3D printing. It is relatively simple and convenient as compared to wax printing and plasma etching methods [14,15]. In our previous papers, hemi channels with opened bottom were fabricated by double sided 3D printing by reversing the paper [17,18,19]. However, there was a problem that the positions of the channel and reservoir could be misaligned when re-attaching to the plate after reversing the paper. To solve this problem, we improved the printing method of hemi channels with open bottom that can continuously print all structures without paper reversing and alignment through combining side and top barriers and reservoir in single-channel design (Supplementary Material Figure S1). The enclosed channels were fabricated by printing the bottom barrier on the entire bottom of the paper and then reversing the paper to print the hemi channel structure. Since channel and reservoir were printed continuously, alignment was not required (Figure 2c).

### 3.2. Effect of Channel Height on Wicking Speed in Enclosed Channel

The wicking speed of the fluid decreased with height of the channel being decreased (Figure 3c,d, Video S1). Thus, in decreasing the channel height from 0.3 to 0.17 mm, we reduced the wicking distance at 250 s two times from 14 to 7 mm. Our vat photopolymerization method had the greater advantage of being able to fabricate channels with various wicking speeds simply by controlling the exposure time of the top barrier. It is comparatively easy to control the wicking speed over the conventional methods that need to change the channel geometry (width, length, and resistance structure) on the whole [23,24]. For glucose detection, 0.3 mm height of enclosed channel with the fastest wicking speed was used to shorten the time required for detection.

### 3.3. Wicking Speed in Hemi and Enclosed Channels Wetted with Sufficient Sample Volume

**Figure 4:**
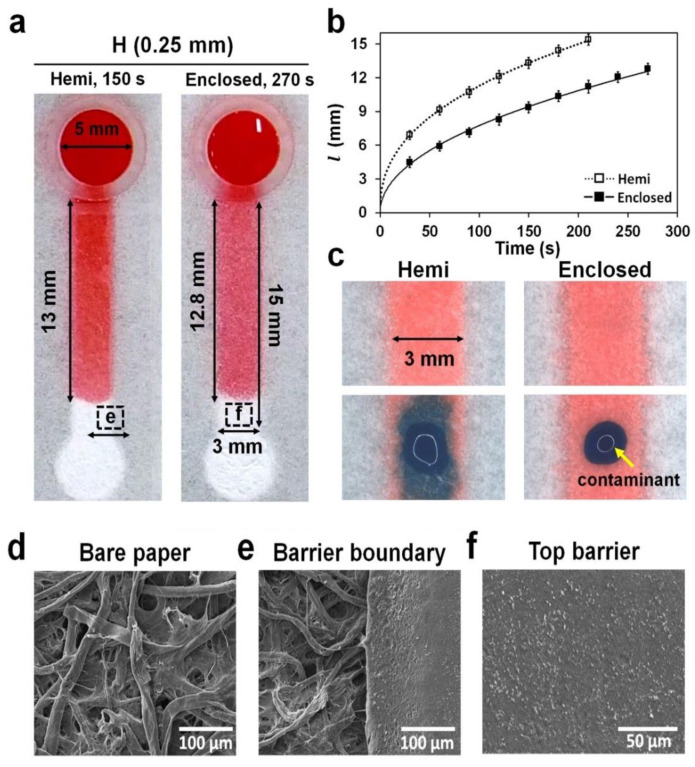
Wicking and contamination in hemi and enclosed channels. (**a**) The top view of both channels stained with 30 μL of a red dye solution from the reservoir in the 3D-μPADs. The images of hemi and enclosed channels (15 mm long) were taken at 150 and 270 s, respectively, when the solution head traveled approximately 13 mm from the reservoir. Red dye in the enclosed channel was also visible, such as the one in the hemi channel, because the top barrier in the enclosed channel was semi-transparent. (**b**) The wicking distance (*l*) of the dye solution was measured at different times (0–270 s) in the hemi channel (empty square) and the enclosed channel (square). *n* = 3. (**c**) Dispersion of a blue dye solution (1 μL) dropped onto the top of both channels, simulating contamination of the channel. (**d**–**f**) SEM images: (**d**) the bare paper, (**e**) barrier boundary in the hemi channel (**a**), and (**f**) top barrier in the enclosed channel (**a**).

To understand the difference in wicking properties between hemi and enclosed channels, we dropped 30 μL of a red dye solution into the reservoir of hemi and enclosed channels with same channel dimensions (15 mm × 3 mm × 0.25 mm; *L* × *W* × *H*) and their wicking speeds were compared (Figure 4a and Figure S2c). It took approximately 150 and 270 s to reach a distance of 13 mm from the reservoir in the hemi and enclosed channels, respectively. The wicking distance (*l*) profiles showed that the wicking speed in the hemi channels was comparatively higher than that in the enclosed channels (Figure 4b). It is known that hydrophobic barriers reduce fluid velocity by inducing a hydrophobic drag force in the fluid flowing through the channel [25]. Unlike hemi channels, which induce the drag force in the bottom and side barriers, enclosed channels induce the drag force in all barriers, including the top, thereby significantly reducing the fluid velocity.

### 3.4. Contamination in Hemi and Enclosed Channels

To test if the contamination can be prevented in the enclosed channels, we placed 1 μL of blue ink outside the hemi and enclosed channels wicked with red ink, and their mixing was monitored. As shown in Figure 4c, the blue ink outside the enclosed channels was not mixed up with red ink in the channels until 2 h, unlike in the hemi channels. This result showed that the entry of the blue ink into the channel was prevented because the channel was surrounded by the photocurable resin (Figure 4f), which is off hydrophobic moiety.

### 3.5. Evaporation in Hemi and Enclosed Channels Wetted with Small Volume Samples

**Figure 5:**
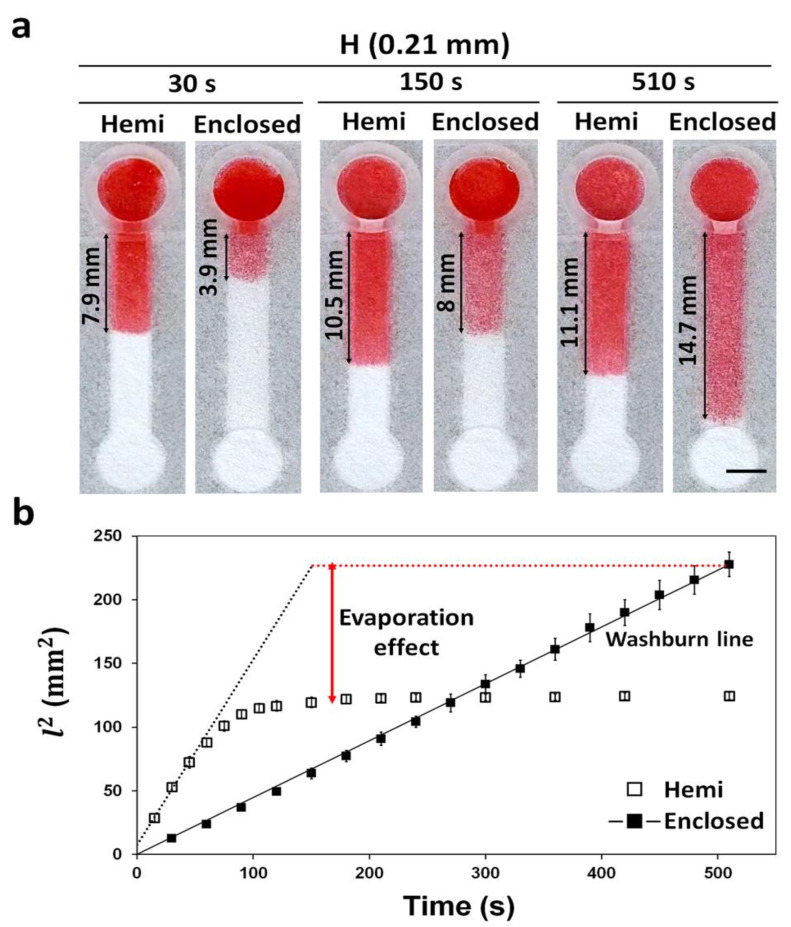
Wicking distance and evaporation effect in hemi and enclosed channels with the same height (0.21 mm) during the time (0–510 s). (**a**) Wicking of a red dye solution (10 μL) in the channels at 30, 150, and 510 s. Scale bar: 3 mm. (**b**) Different wicking speed profiles over time explaining the difference in evaporation between the hemi and enclosed channels. The Washburn line was obtained by the Lucas–Washburn equation (Equation (1)) [27]. The experiment was carried out at 28 °C and a relative humidity of 41%. *n* = 3.

Evaporation of a sample solution during the wicking phenomenon seems to happen throughout the channel, either decreasing the volume of the solution to reach or stopping without reaching the testing zone, thereby impairing the detection [7]. To demonstrate the solution in restricting the sample evaporation using enclosed channels, we placed 10 μL of red dye solution into the reservoirs of hemi and enclosed channels with the same dimensions (15 mm × 3 mm × 0.21 mm; *L* × *W* × *H*), and their wicking distances were compared as a function of time. At 30 and 150 s, the sample solution in the hemi channels traveled faster than enclosed channels (Figure 5a). At 510 s, however, it stopped traveling for the hemi channel, while the sample solution in the enclosed channels reached the end (Video S2). The slow wicking and stoppage of the solution in the hemi channels could be attributed to the evaporation process. Similar results for hemi channels were observed in other studies [8,26].

The difference in the wicking behavior among the hemi and enclosed channels by evaporation function could be compared using the Lucas–Washburn equation (Equation (1)), which describes the behavior of a fluid flowing through a porous media facilitated via capillary reaction [27].

The modified form of the Lucas–Washburn equation ignoring evaporation is as follows:

(1) $$l=\alpha \sqrt{\frac{D\gamma \cos\theta }{4\mu }t},$$

where *l* is the wicking distance, *α* is the drag effect by hydrophobic barrier, t is the wicking time, *γ* is the surface tension, *θ* is the contact angle of water with paper, μ is the viscosity, and *D* is the pore diameter [23]. Therefore, in the case of nil evaporation in the channel, the graph of *l*^2 vs. *t* would show a linear slope [13,26]. As the slope of the enclosed channel is linear, the wicking in the channel follows the Washburn equation thereby confirming near to zero evaporation (Figure 5b). For the hemi channels, the initial wicking (≈60 s) profile followed the Washburn line. However, after 60 s, the profile did not follow the Washburn line (Figure 5b), suggesting that some of the sample solution was lost in the hemi channels via evaporation. As the enclosed channels were surrounded by hydrophobic barriers, the evaporation of the sample solution was prevented. In case of enclosed channels fabricated by wax printing and plasma etching, they were unable to block the pores in paper, requiring additional sealing with tape to completely prevent evaporation [8,13]. The enclosed channels made here by vat photopolymerization printing can completely block the pores of the paper, requiring no additional sealing steps, thereby making the fabrication process simpler. Our enclosed channel fabrication method could be plausibly utilized to fabricate electrochemical paper-based microfluidic devices (ePADs) [28] and paper-based cell culture platform [29] where evaporation is a critical problem. Sample evaporation issue in POCT can be addressed by fabricating μPADs with enclosed channels.

### 3.6. Improvement of Sensitivity for the Detection of Glucose in Enclosed Channels through Inhibition of Evaporation When Dealing with Small Volume Samples

**Figure 6:**
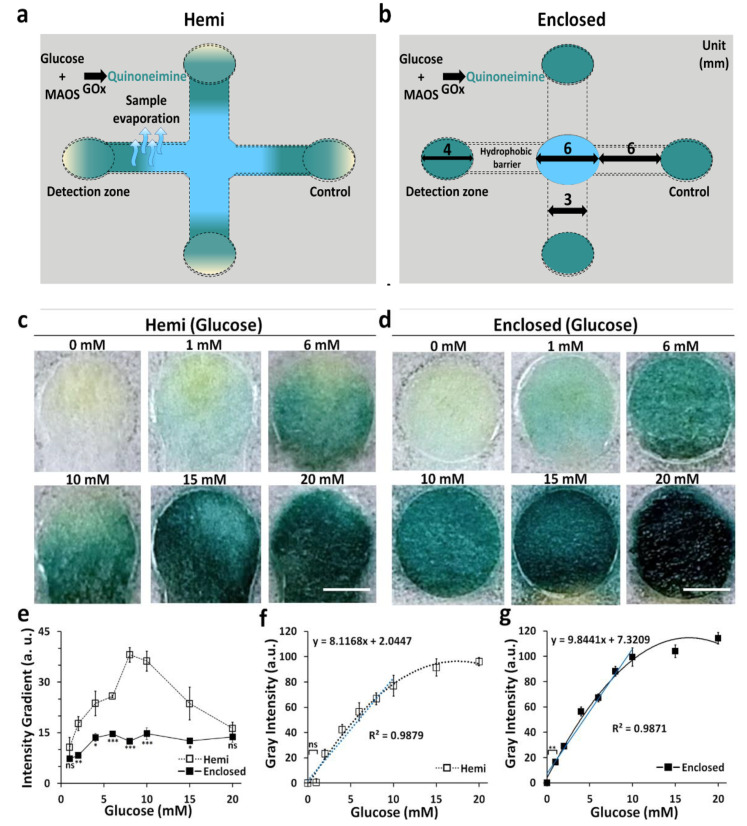
Comparison of sensitivity and uniformity of colorimetric detection of glucose in hemi channels and enclosed channels. (**a**,**b**) Schematics of the 3D-μPADs with hemi (**a**) and enclosed (**b**) channels for detection of glucose. (**c**,**d**) Representative images of the detection zones reacted with various glucose concentrations (0–20 mM) in hemi (**c**) and enclosed (**d**) channels (scale bar: 2 mm). (**e**) Color intensity gradient as a function of different concentrations of glucose in hemi channels and enclosed channels. *n* = 3, * *p* < 0.05, ** *p* < 0.01, *** *p* < 0.001. (**f**,**g**) Calibration curves of glucose were calculated from obtained images with various concentrations in hemi (**f**) and enclosed (**g**) channels. *n* = 3, ** *p* < 0.01.

Evaporation of the sample during transport lowers the sample delivery efficiency, which lowers the detection sensitivity of the POCT [7]. To test the evaporation prevention efficacy of the enclosed channel, we used the channels holding the same height (0.3 mm) for both hemi and enclosed channels to colorimetrically detect glucose at different concentrations (0–20 mM) and compared them in terms of sensitivity (Figure 6a,b). Color in the detection zone of the enclosed channels seemed to be more uniform than that of the detection zone observed in the hemi channels **(**Figure 6c,d). The difference in uniformity was verified by analyzing the color gradients across the detection zones in both hemi and enclosed channels. The values of the color gradients in the channels were analyzed quantitatively, and the color gradient values were compared by converting the images of the detection zone into grayscale. The approximate color gradient values of hemi channels and enclosed channels ranged from 10 to 40% and 7 to 15%, respectively, signifying that the color across the detection zone for enclosed channels was more uniform than hemi channels (Figure 6e). LODs for glucose calculated by dose–response curve for hemi and enclosed channels were 1.1 and 0.3 mM, while the coefficient of variation (CV) values for hemi and enclosed channels were 15.7% and 9.1%, respectively. In the case of detecting 1 mM glucose, the change in gray intensity could not be observed visually for μPADs using the hemi channel, whereas detection was significantly possible using enclosed channels (Figure 6f,g). These performance indices for the detection (Table 2) showed that the enclosed channels were comparatively advantageous over hemi channels in terms of sensitivity and reproducibility for the detection of glucose (Figure 6f,g).

High values in the color gradients of hemi channels could be attributed to the sample evaporation during wicking, therein limiting the transportation of glucose to the detection zone. As a result, the distribution of glucose in the detection zone might be inhomogeneous and generate non-uniform discrimination with low-intensive colors across the detection zones. Similar results have been reported for an open-type μPADs system using a very small volume sample [30]. In contrast, evaporation of such sample solution was suppressed, resulting in uniform transportation of glucose across the detection zone, which generated high-intensity and uniform colors (Video S3). In a limited volume of sample, the evaporation of a sample solution in μPADs can cause reduction in the sensitivity. Our results suggest that 3D μPADs with enclosed channels can be used to improve the LOD in POCT, especially in cases where a small volume sample is used. Indeed, previous work with hemi channels used 500 μL of sample volume to obtain a uniform color intensity [17], but using the enclosed channel allowed us to obtain a uniform color intensity with 20 μL of a small volume sample.

In summary, the enclosed channel fabrication method using vat photopolymerization has significant advantages over conventional devices: (1) It is easy to fabricate and modify the channel design as no etch-mask or photo-mask were used during the fabrication process. (2) The height of the channel can be precisely controlled via the UV exposure method, and it possesses higher resolution than the wax printing method. (3) It is suitable for long-term storage because there is no risk of channel damage, which generally arises out from melting of wax and adhesive. (4) The enclosed channel prevents the evaporation of a small volume sample during transport, thereby improving the sensitivity level and color uniformity without additional sealing. (5) The wicking rate can be controlled by adjusting the height of the channel, thus preventing sample loss from dead zones of long channels. (6) The light-transmitting resin allowed us to visualize the movement of the fluid flow inside the enclosed channel.

## 4. Conclusions

This is the first time that vat photopolymerization has been used to fabricate enclosed channels on single-filter paper through simple double-sided printing. Enclosed channels with an appropriate wicking speed can be fabricated by adjusting the height of the channel by varying the exposure time. The enclosed channels fabricated by our method can prevent the sample evaporation without any additional sealing agent, allowing us to handle a small volume sample in 3D-μPADs with enhanced sensitivity and color uniformity compared with other similar devices. Through the use of a 3D-printed enclosed channel, colorimetric detection of various biomarkers in patient samples will be conducted as a future work. Furthermore, by combining the 3D printed enclosed channel and the electrochemical sensor, we intend to develop a sensor that can monitor biomarkers included in a small volume sample in real time. This may be used for patients who cannot collect large amounts of blood, such as neonatal diabetes.

## Figures and Tables

**Table 1:** Vat photopolymerization parameters for enclosed channel fabrication.

Parameters	
Power (mW/cm^2^)	2.8
Printing plate size (mm × mm)	96 × 54
Layer thickness (mm)	0.1
Motor speed (mm/s)	1
Motor speed adjustable height (mm)	2
Initial layer waiting time (s)	6
Layer waiting time (s)	4
Exposure time for bottom barrier (s)	1
Exposure time for side barrier (s)	8
Exposure time for top barrier (s)	1–4

**Table 2:** Comparison of glucose detection results of hemi channel and enclosed channel. To maximize the evaporation effect, we used 20 μL of PBS containing glucose at various concentrations (0–20 mM).

Glucose Detection in a Small Volume Sample (20 μL)		
	Hemi Channel	Enclosed Channel
Coefficient of variation (CV)	15.7%	9.1%
Range of color gradient ^┴^	10–40%	7–15%
Limit of detection (LOD) *	1.1 mM	0.3 mM

* The LOD was calculated by multiplying the standard deviation of the negative control by 3 [18,22]. ^┴^ A standard deviation of the gray intensity in the detection zone was used to quantify the color gradient [21].

## Data Availability

The data presented in this study are available in the article or Supplementary Material.

## Supplementary Materials

The following are available online at https://www.mdpi.com/article/10.3390/diagnostics11060977/s1, Figure S1: 3D design and fabrication method of hemi channels. (a) An improved 3D design to print the hemi channels with an open bottom at once without paper reversing and alignment. It is a structure that combines side and top barriers and a reservoir. (b) Schematic describing vat photopolymerization printing steps to fabricate an hemi channel in filter paper. The paper was soaked with the UV-curable resin polyurethane before initiating printing steps (i-iii). Exposure times for the side, top barriers, and the reservoir were 8, 1–4, and 20 s, respectively. Washing and drying steps (iv) were performed outside the printer. Figure S2: The height of hemi channels in filter paper at different UV exposure times (1–9 s). (a) Cross-sectional views of hemi channels stained with red dye flowed from the reservoir. At 1 s, the filter paper maintained its hydrophilicity and was completely stained with the dye, except the bottom part that was exposed. At 9 s, the paper was completed crosslinked with the resin and not stained with the dye. (b) Hemi channels with different heights by varying UV exposure times were used to print the bottom barrier in the paper, whereas their side barriers were printed with UV exposure at 8 s. n = 3. (c) l vs. time curve of water wicking along hemi channels with different heights (0.17 to 0.3 mm). A total of 30 μL of red dye was flowed from the reservoir that was connected to the channel. n = 3, scale bar: 150 μm. Figure S3: Measurement of gray intensity value of red dye according to channel location in hemi channel and enclosed channel. (a) Since evaporation occurred on all surfaces in the hemi channel, a large amount of red dye was placed in the channel, and the channel showed a high gray intensity value. (b) Since evaporation occurred only in the inlet and detection zone in the enclosed channel, a large amount of red dye was transported to the detection zone, which showed a high gray intensity value. Scale bar: 5 mm. Table S1: Channel formation height and wicking speed according to exposure time. To confirm the evaporation prevention effect of the enclosed channel, we flowed a small volume sample (10 μL) through a 15 mm long channel to ensure that the sample reached to the detection zone. Video S1: A video was taken of the wicking of fluid flowing inside an enclosed channel with different heights. Video S2: Video recording of a small volume sample (10 μL) flowing through the hemi channel and the enclosed channel of the same height (0.21 mm) to compare the evaporation of the sample. Video S3: a video was recorded of the color change of the detection area after the glucose sample was completely wetted with μPAD. In the hemi channel, the color moved towards the channel and the intensity of the detection area decreased, whereas in the enclosed channel, the color moved to the detection area and became darker.
