# Supplementary material for: Enhanced Sensing Behavior of Three-Dimensional Microfluidic Paper-Based Analytical Devices (3D-μPADs) with Evaporation-Free Enclosed Channels for Point-of-Care Testing
Source: Diagnostics (Basel). 2021 May 28;11(6):977. doi: 10.3390/diagnostics11060977 (PMC8229230; doi:10.3390/diagnostics11060977)
Supplement: Supplementary file 1 [file diagnostics-11-00977-s001.zip › Diagnostics_Supplementary information_JH.docx]

**Diagnostics**

**Supplementary information**

**Title: Enhanced sensing behavior of three-dimensional microfluidic paper-based analytical devices (3D-μPADs) with evaporation-free enclosed channels for point of care testing**

Jaehyung Jeon^1^, Chanyong Park^1^, Dinesh Veeran Ponnuvelu^1^ and Sungsu Park^1,2,3*^

^1^ School of Mechanical Engineering, Sungkyunkwan University, Suwon, 16419, Korea

^2^ Department of Biomedical Engineering, Sungkyunkwan University, Suwon, 16419, Korea

^3^ Institute of Quantum Biophysics (iQB), Sungkyunkwan University, Suwon, 16419, Korea

^*^ CORRESPONDING AUTHOR: S. Park: School of Mechanical Engineering, Sungkyunkwan University, Seoburo 2066, Jangan-gu, Suwon, 16419, Korea

**
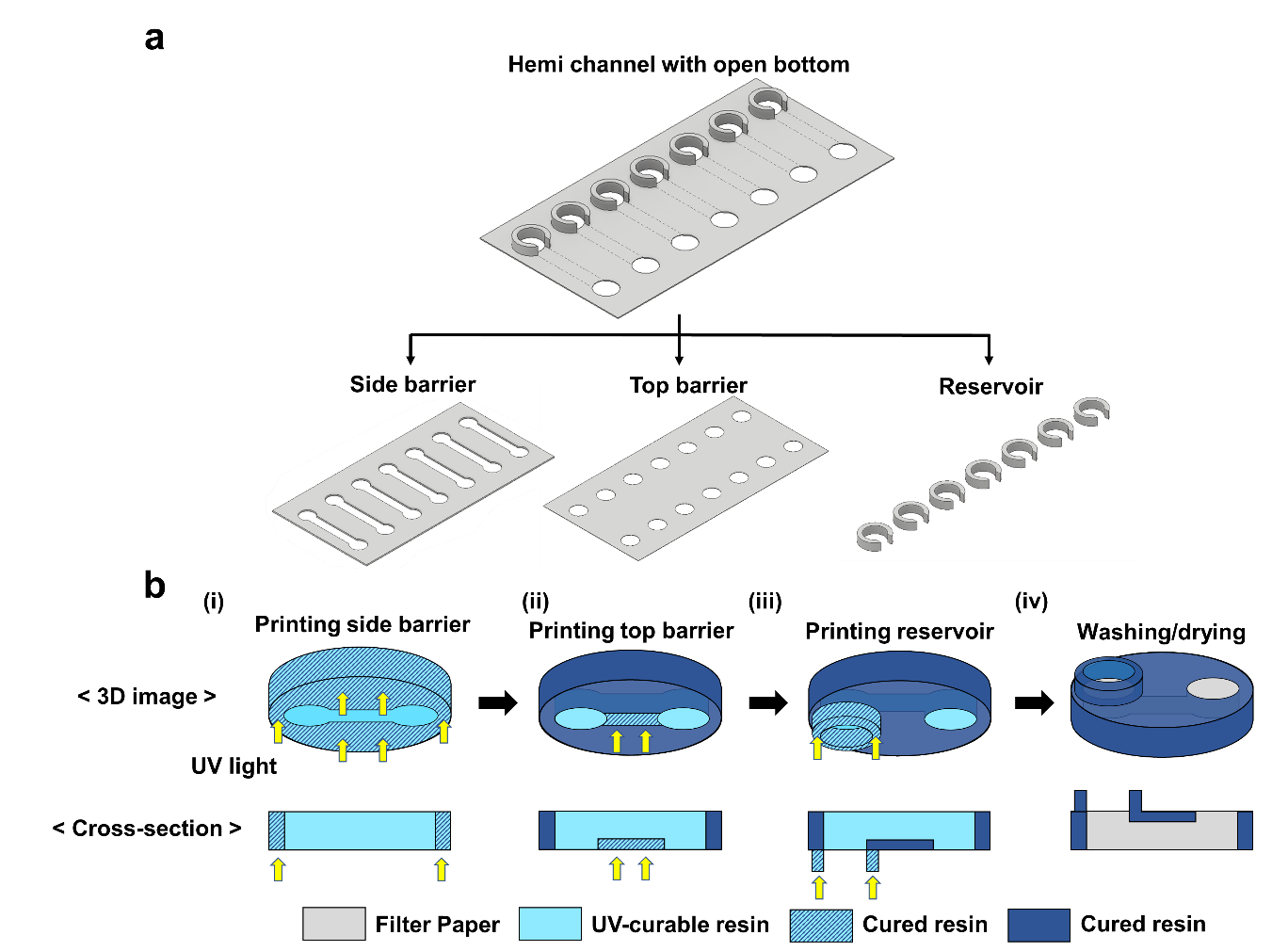
 Figure S1:** 3D design and fabrication method of hemi channels. (a) An improved 3D design to print the hemi channels with an open bottom at once without paper reversing and alignment. It is a structure that combines side and top barriers and a reservoir. (b) Schematic describing DLP 3D printing steps to fabricate an hemi channel in filter paper. The paper was soaked with the UV-curable resin polyurethane before initiating printing steps (i-iii). Exposure times for the side, top barriers, and the reservoir were 8, 1-4, and 20 s, respectively. Washing and drying steps (iv) were performed outside the printer.


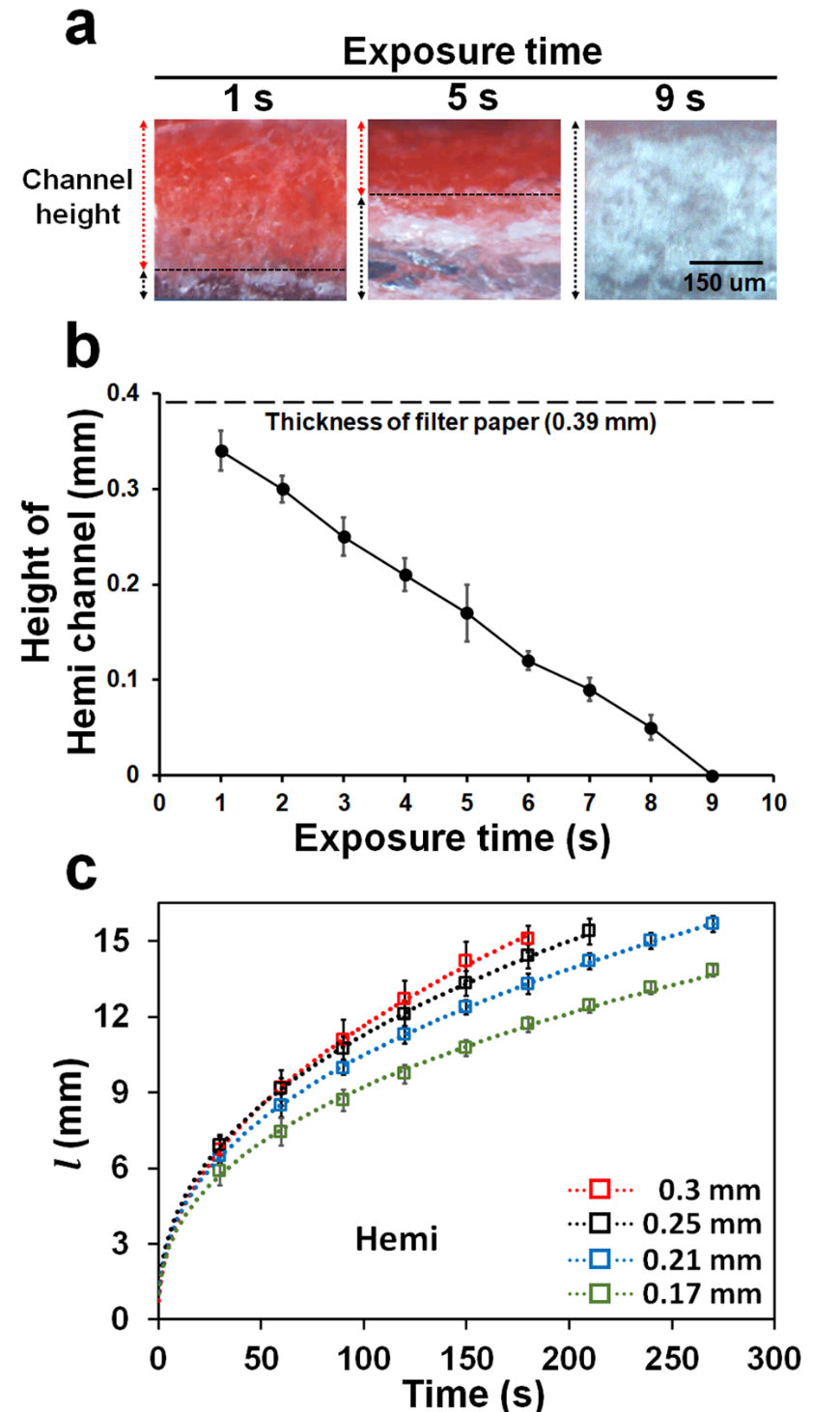


**Figure S2:** The height of hemi channels in filter paper at different UV exposure times (1-9 s). (a) Cross-sectional views of hemi channels stained with red dye flowed from the reservoir. At 1 s, the filter paper maintained its hydrophilicity and was completely stained with the dye except the bottom part that were exposed. At 9 s, the paper was completed crosslinked with the resin and not stained with the dye. (b) Hemi channels with different heights by varying UV exposure times to print the bottom barrier in the paper whereas their side barriers were printed with UV exposure at 8 s. n = 3. (c) $l$ vs time curve of water wicking along hemi channels with different heights (0.17 mm to 0.3 mm). 30 μl of red dye was flowed from the reservoir that was connected to the channel. n = 3, scale bar: 150 μm.


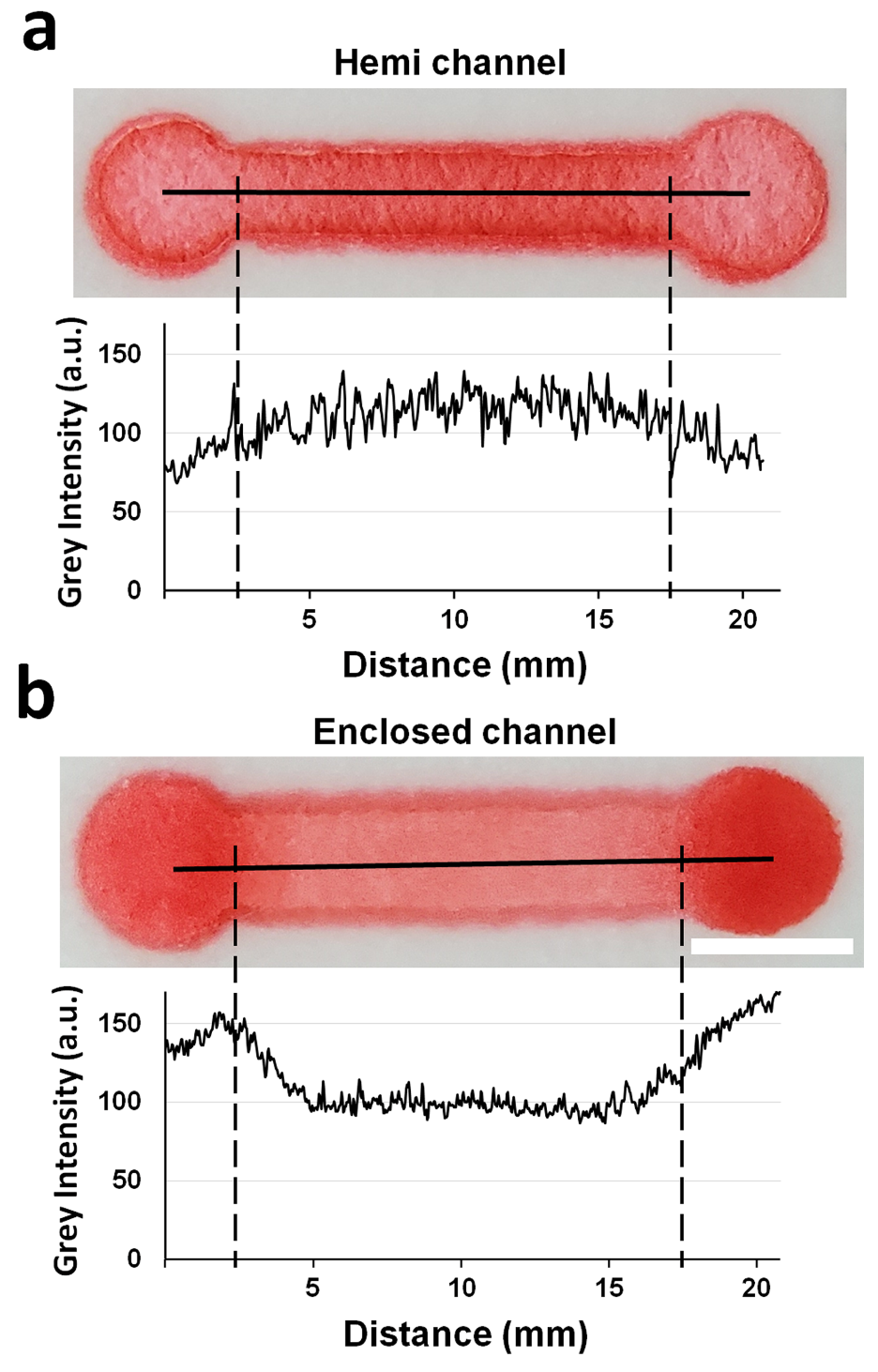


**Figure S3:** Measurement of gray intensity value of red dye according to channel location in hemi channel and enclosed channel. (a) Since evaporation occured on all surfaces in the hemi channel, a large amount of red dye was placed in the channel, and the channel shows a high gray intensity value. (b) Since evaporation occured only in the inlet and detection zone in the enclosed channel, a large amount of red dye was transported to the detection zone which showed a high gray intensity value. scale bar: 5 mm.

**Supplementary Video:**

**Video S1:**

movie was taken of wicking of fluid flowing inside an enclosed channel with different heights.

**Video S2:**

movie recorded a small sample volume (10 L) of fluid flowing through the hemi channel and the enclosed channel of the same height (0.21 mm) to compare the evaporation of the sample.

**Video S3:**

movie recorded of the color change of the detection area after the glucose sample was completely wetted with μPAD. In the hemi channel, the color moves towards the channel and the intensity of the detection area decreases, whereas in the enclosed channel, the color moves to the detection area and becomes darker.
